# Interventions to Improve Outcomes After Pregnancy Loss: A Systematic Review

**DOI:** 10.1111/1471-0528.70043

**Published:** 2025-10-17

**Authors:** Becky MacGregor, Samuel Madejowski, Helen Leach, Clare Macdonald, Jeremy Dale, Amy Grove, Alexander E. P. Heazell, Jo Parsons, Sophie Staniszewska, Sarah Hillman

**Affiliations:** ^1^ Applied Health Sciences University of Birmingham Birmingham UK; ^2^ Warwick Medical School University of Warwick Coventry, West Midlands UK; ^3^ Warwick Applied Health University of Warwick Coventry, West Midlands UK; ^4^ School of Social Policy and Society University of Birmingham Birmingham UK; ^5^ Maternal and Fetal Health Research Centre University of Manchester Manchester UK

**Keywords:** bereavement counselling, miscarriage, puerperium, recurrent, systematic reviews

## Abstract

**Background:**

Stillbirth, second‐trimester miscarriage and recurrent miscarriage carry significant consequences for women. We lack sufficient high‐quality evidence of interventions to improve a woman's health and subsequent pregnancy outcomes after discharge to the community.

**Objective:**

Assess the effectiveness of interventions to improve general health and subsequent pregnancy outcomes for non‐pregnant women who have had a stillbirth, second trimester miscarriage, or recurrent miscarriage.

**Search Strategy:**

Database searches were undertaken in August 2022 (updated March 2024) and limited to full‐text documents published from 1995.

**Selection Criteria:**

Interventions delivered after discharge for non‐pregnant women following a pregnancy loss.

**Data Collection and Analysis:**

Screening was performed independently by two reviewers; narrative synthesis was undertaken. Risk of bias was assessed by RoB‐2, ROBINS‐I or the Mixed Methods Appraisal Tool.

**Main Results:**

A total of 18 603 abstracts screened; 196 full texts assessed and 15 papers included. The quality of evidence was low, and the primary aim of the review was not met due to limited evidence. All included studies aimed to improve mental health. No studies were identified that aimed to improve the physical health of women or subsequent pregnancy outcomes.

**Conclusions:**

There is a significant evidence gap regarding how best to care for women who experience pregnancy loss after discharge to the community. There is an urgent need for research to determine which interventions are most effective to improve a woman's short‐ and long‐term health and subsequent pregnancy outcomes following a stillbirth, second‐trimester miscarriage or recurrent miscarriage.

**Trial Registration:**

PROSPERO Registration: CRD42022360264

## 1 Introduction

Stillbirth, second trimester miscarriage and recurrent miscarriage are distressing life events that carry significant consequences for women, their families and society [1, 2]. Women who experience a stillbirth have a nearly five‐fold increased risk of stillbirth in a subsequent pregnancy [3]. Women who have experienced a second trimester miscarriage are at 10 times higher risk of a subsequent second trimester miscarriage [4]. Experiencing a pregnancy loss puts women at increased risk of adverse outcomes in future pregnancies, including pre‐term delivery, pre‐eclampsia and low‐birthweight infants [1, 5, 6]. Having a stillbirth or miscarriage is predictive of longer‐term health complications, including cardiovascular disease [1, 7]. Obesity, smoking and poorly controlled diabetes or hypertension are all modifiable risk factors [1, 5, 8].

There is minimal guidance on how to deliver care following pregnancy loss in the community [9, 10] and significant variation in practice [11]. Guidelines from the Royal College of Obstetricians and Gynaecologists recommend an appointment in secondary care following a stillbirth and that families are offered counselling, but the evidence grade for these recommendations is low [9]. Women who experience recurrent miscarriage should be referred to a specialist miscarriage clinic [12] and there is currently no specific guidance for care after a second trimester miscarriage. The UK's National Bereavement Care Pathway recognises good community care after discharge is a key component of care following a pregnancy loss, but makes no specific recommendation on what care should consist of [13]. We currently lack sufficient high‐quality evidence of interventions that may be used to improve a woman's health and subsequent pregnancy outcomes following stillbirth, second trimester miscarriage, or recurrent miscarriage after discharge into the community.

This systematic review aims to answer the following questions:

1. How effective are interventions aimed at improving general health and subsequent pregnancy outcomes for non‐pregnant women who have had a stillbirth, second trimester miscarriage or recurrent miscarriage?
2. What are the experiences of women who have ever had a stillbirth, second trimester miscarriage or recurrent miscarriage regarding acceptability of interventions and barriers to uptake?
3. What are the experiences of healthcare professionals delivering interventions to improve a woman's health and subsequent pregnancy outcomes?

This review refers to women and women's health; however, the concepts herein apply to all birthing people. We acknowledge the importance of providing equitable care for all women and birthing people.

## 2 Methods

The review protocol was registered on the International Prospective Register of Systematic Reviews (CRD42022360264) [14]. The Preferred Reporting Items for Systematic Reviews Protocol and Meta‐Analyses guidelines were followed [15].

There was no direct patient or public involvement or engagement (PPIE) in the review; however, the topic for the review was informed by a previous PPIE project exploring community‐based care provision after late pregnancy loss [16].

### 2.1 Inclusion and Exclusion Criteria

The following definitions were used:

*Stillbirth*: the legal definition of stillbirth in the country in which the study was conducted.

*Second trimester miscarriage*: a spontaneous loss of pregnancy between 13 + 0 and 23 + 6 weeks' gestation [12].

*Recurrent miscarriage*: two or more consecutive spontaneous pregnancy losses < 24 weeks gestation [1, 17]. This definition captures the international variation in terminology [1].

The PICO framework [18] was used to define the review question and inclusion and exclusion criteria (Table 1). There were no relevant core outcome sets available for outcome reporting. A core outcome set for stillbirth research is being developed by the iCHOOSE collaborative [19] and for mental health following early pregnancy loss by the COSMEP collaborative [20].

**TABLE 1:** PICO question development.

PICO	Inclusion criteria	Exclusion criteria
**P**opulation	Women who have ever had a stillbirth, second trimester miscarriage or recurrent miscarriage	Women in their first pregnancy or who have never had a stillbirth, second trimester miscarriage or recurrent miscarriage
**I**ntervention	Non‐surgical interventions that have been initiated in the interpregnancy interval or following a pregnancy loss i.e., when a woman is not pregnant Classified into preventative and therapeutic, and may be pharmacological, nutritional, behavioural, psychological, educational or environmental Interventions that occur after discharge from the initial hospital stay or episode of care relating to the pregnancy loss	Interventions that occur antenatally, intrapartum or prior to a first pregnancy Interventions that occur prior to initial hospital discharge following a stillbirth, second trimester miscarriage or miscarriage Surgical interventions
**C**omparator	Women who have ever had a stillbirth, second trimester miscarriage or recurrent miscarriage who have not been exposed to the intervention.	Women who have ever had a stillbirth, second trimester miscarriage or recurrent miscarriage who have been exposed to the intervention
**O**utcomes	Change in the incidence of Stillbirth in the subsequent pregnancy Second trimester miscarriage in the subsequent pregnancy Successful pregnancy following recurrent miscarriage Pre‐term birth in a subsequent pregnancy Placental abruption in a subsequent pregnancy Low birthweight in a subsequent pregnancy Hypertensive disorders of pregnancy Gestational diabetes Maternal depression or anxiety Conception rates The experiences of women, who have ever experienced stillbirth, second trimester miscarriage or recurrent miscarriage of participating in an intervention to improve maternal and fetal outcomes in a subsequent pregnancy. The experiences of healthcare professionals of delivering an intervention to improve maternal and fetal outcomes in a subsequent pregnancy.	

### 2.2 Information Sources

Searches were undertaken of MEDLINE, EMBASE, PUBMED, CINAHL, Web of Science and Psychinfo on 18th August 2022 (updated 13th March 2024). Reference lists from included studies were searched for relevant papers, and reference lists from relevant systematic reviews [21, 22, 23, 24, 25]. Grey literature was reviewed from national bodies, including the National Institute for Health and Care Excellence, Scottish Intercollegiate Guidelines Network and medical colleges in Australia, Canada, New Zealand, the UK and the USA; ten guidelines were identified for inclusion [12, 26, 27, 28, 29, 30, 31, 32, 33, 34].

### 2.3 Search Strategy

The search was limited to studies published in English and from 1995 onwards to ensure relevance to current clinical practice. No restrictions were placed on setting. All types of primary empirical study were eligible; case studies, case reports, discussion pieces, editorials, commentaries and systematic reviews were excluded. The search strategy was piloted (Appendix S1).

### 2.4 Screening and Data Collection

Covidence software was used to collate abstracts and remove duplicates [35]. Titles and abstracts were screened independently by two reviewers (BM and HL or CM or SM); a third reviewer (SH) screened titles and abstracts where there was disagreement. Full text papers were read by two reviewers (BM and SM); a third reviewer (SH) reviewed papers to achieve consensus where required. A standardised data extraction document was designed and piloted (BM and SM) to extract data. Data were extracted by two reviewers independently (BM and SM). Extracted data included year of publication, setting, country, study design, participant characteristics, type of pregnancy loss, details of intervention and outcomes. Where available qualitative data were extracted describing the views and perceptions of women and health care professionals.

### 2.5 Quality Assessment Findings

Risk of bias assessment was undertaken by two reviewers (BM or SH), using RoB‐2 [36], Robins‐I [37] or MMAT [38] depending on study design (Appendix S2); 10% were crosschecked for concordance.

### 2.6 Synthesis

A meta‐analysis was planned to assess the effectiveness of the interventions on the primary outcome: change in the incidence of pregnancy loss in a subsequent pregnancy. Due to the heterogeneity of reported outcome measures, this was not feasible; a narrative synthesis was conducted for all data types.

## 3 Results

### 3.1 Included Studies

**FIGURE 1:**
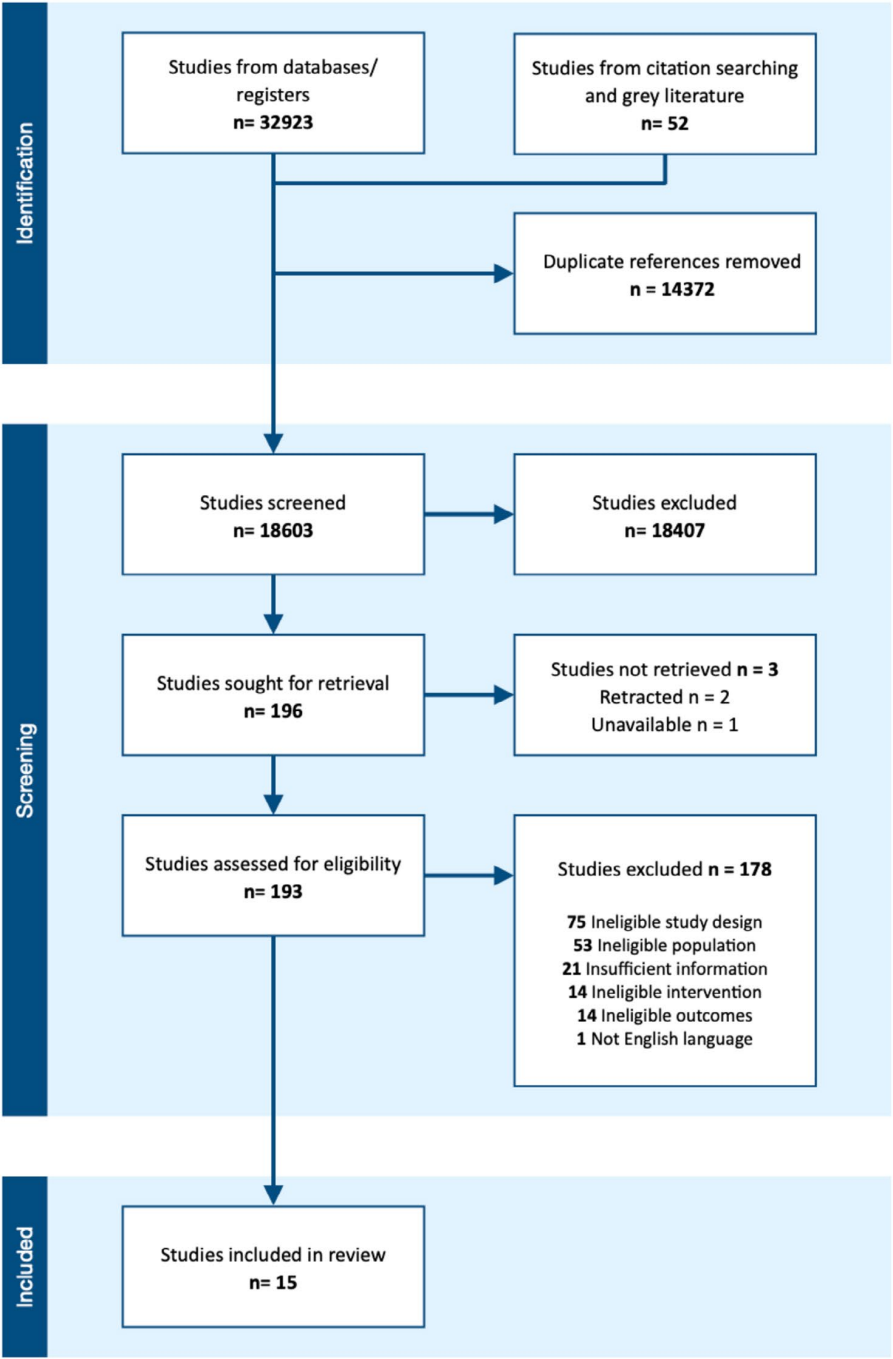
Prisma diagram.

A total of 18 561 documents were identified for abstract screening from database and grey literature searches (following de‐duplication). 41 documents were identified through citation searching. 196 full‐text articles were identified for retrieval. See PRISMA diagram (Figure 1).

Reasons for exclusion are listed in the PRISMA diagram. Studies including mixed populations which were not separated into subgroups of interest were excluded, as were those where the intervention started in the interpregnancy interval but continued antenatally.

15 studies were eligible for inclusion; these were published between 2001 and 2023 and included 826 participants across 14 studies. Two papers reported on one pilot study with 12 month follow up [39, 40]. A summary of included studies is presented in Tables S1 and 2. Three studies self‐classified as pilot studies [39, 40, 41, 42], three as randomised control trials [43, 44, 45], two as randomised feasibility trials [46, 47] and the remainder were a mix of semi‐experimental study designs and surveys [48, 49, 50, 51, 52, 53]. Participant numbers ranged from 14 to 103. Study settings included India, Iran, Japan, Taiwan, UK and USA. Seven interventions were set in the community [39, 40, 41, 46, 47, 51, 52, 53], five in secondary care [42, 43, 44, 45, 48] and two settings were unclear [49, 50].

**TABLE 2:** Summary of interventions and outcome measures.

Study	Intervention	Type of pregnancy loss	Outcome measures
Nakano et al. (2013) [42]	Individual CBT	Recurrent miscarriage	Depression and anxiety scores
Basirat et al. (2022) [45]	Group CBT and Sertraline	Recurrent miscarriage	Depression and anxiety scores
Rowsell et al. (2001) [48]	Individual counselling	Recurrent miscarriage	Depression, anxiety, intrusive thoughts, avoidance and coping scores
Cacciatore et al. (2017) [49]	Counselling	Stillbirth	Views of women of counselling
Navidian et al. (2017) [51]	Group counselling	Stillbirth	Post‐traumatic stress score
Navidian et al. (2018) [52]	Group counselling	Stillbirth	Grief severity score
Chang et al. (2021) [44]	Individual counselling	Recurrent miscarriage	Perceived stress, sleep quality, depression and perceived social support scores
Roberts et al. (2015) [39] Roberts et al. (2016) [40]	Group mindfulness	Stillbirth	Mindfulness, life satisfaction, perinatal grief, depression and perceived social support scores
Roberts et al. (2016) [41]	Group mindfulness	Stillbirth	Mindfulness, life satisfaction, perinatal grief, depression and perceived social support scores
Huberty et al. (2020) [46]	Online yoga videos	Stillbirth	PTSD symptoms
Cacciatore et al. (2007) [50]	Support groups	Stillbirth	Traumatic stress response score
Beck et al. (2019) [53]	Online archive	Stillbirth	Frequency of reported emotions
Sullivan et al. (2022) [47]	Facebook group	Stillbirth	Feasibility. Traumatic stress response, depression, anxiety and support seeking scores.
Hung et al. (2023) [43]	Website	Recurrent miscarriage	Perceived stress, sleep quality, depression and perceived social support scores

Five interventions were implemented in populations of women who had recurrent miscarriage [42, 43, 44, 45, 48] and nine in populations of women who had a stillbirth [39, 40, 41, 46, 47, 49, 50, 51, 52, 53]. All interventions had a primary aim to improve mental health; no interventions were identified that addressed physical health, long‐term health, or subsequent pregnancy outcomes. Nine studies reported on women's experiences of participating in interventions [39, 40, 41, 43, 45, 46, 47, 49, 50, 53]. No studies reported on the experiences of healthcare professionals.

### 3.2 Quality Assessment Findings

Overall, the risk of bias was high; only two studies were assessed as low risk. Ten studies were assessed as at high, serious, or critical risk of bias (Appendix S2). No studies were excluded based on methodological quality. The studies were considered for GRADE assessment [54], but heterogeneity of outcome reporting and variability in study design meant this wasn't possible. As all the studies had small sample sizes, most studies were at high risk of bias and most study designs were non‐randomised; the overall quality of evidence was low.

### 3.3 Results of Synthesis

Fourteen interventions were included, reported across 15 papers. The interventions were classified as Cognitive Behavioural Therapy (CBT) and/or anti‐depressants, counselling, mindfulness techniques including yoga, support groups and website‐based interventions.

### 3.4 CBT and/or Anti‐Depressants

Two studies looked at CBT and/or anti‐depressants for women with recurrent miscarriage. One study examined individual CBT in a secondary care setting [42], the other examined group CBT in a community setting vs. Sertraline (a selective serotonin reuptake inhibitor) vs. routine care [45].

Both studies found CBT significantly reduced depression scores at the end of the intervention (mean Beck Depression Inventory Score 13.6 (SD, 8.2) at baseline versus 5.2 (SD, 4.4) post intervention (*p* = 0.001)) [42] (mean Beck Depression Inventory Score 23.1 (SD 9.89) at baseline versus 13.4 (SD 12.2) post intervention) [45]. The evidence for reduction in anxiety scores with CBT was conflicting, with a significant reduction found with individual CBT (mean State–Trait Anxiety Inventory‐state Score 49.0 (SD, 7.1) at baseline versus 38.0 (SD, 10.2) post intervention (*p* = 0.016)) [42] but not with group CBT [45].

Sertraline significantly reduced anxiety and depression scores at the end of the intervention [45] (Table S1), but this reduction did not persist for depression at the 3‐month follow up [45].

Both studies had 20 or fewer participants exposed to each intervention. The limited evidence suggests CBT and Sertraline may be effective in reducing anxiety and depression scores in women with recurrent miscarriage, but study design limitations and small sample size affect validity.

### 3.5 Counselling

Five interventions used counselling [44, 48, 49, 51, 52]; all had small numbers of participants (*n* = 37–103) and a high risk of bias.

One study was a survey of women who self‐reported engagement with counselling after a stillbirth [49]. Specific details of counselling type were not reported. There was wide variability in responses (Table S1 ), and study limitations make it difficult to draw conclusions.

Four studies were delivered face‐to‐face by trained healthcare professionals [44, 48, 51, 52] and assessed different outcome measures related to mental health and wellbeing [44, 48, 51, 52] (Table 2). Two involved women who had a stillbirth, consisted of group counselling [51, 52] and assessed reduction in post‐traumatic stress score [51] or grief severity score [52]. A significant decrease was seen in both studies (mean Prenatal Posttraumatic Stress Questionnaire 7.22 (SD 4.19) at baseline versus 4.12 (SD 2.14) post intervention (*p* = 0.0001)) [51] mean Grief Severity Score 104.06 (SD 24.00) at baseline versus 86.14 (SD 15.00) post intervention (*p* = 0.0001) [52]. Two involved women who had recurrent miscarriages and consisted of individual counselling sessions delivered face‐to‐face [44, 48]. Both interventions showed significant reductions in depression scores (mean Edinburgh Prenatal Depression Score 9.57 (SD 4.10) at baseline versus (*p* = 0.037)) [44] (mean HADs depression score 5.92 (SD 3.81) at baseline vs. 4.65 (SD 4.15) post intervention (*p* < 0.01)) [48] (Table 2 and S1 for all outcome measures).

The limited evidence suggests that counselling interventions may improve mental health and wellbeing [44, 48, 51, 52]. Due to multiple reporting outcomes and differences in delivery methods it was not possible to determine comparative effectiveness.

#### 3.5.1 Mindfulness Techniques Including Yoga

Three studies investigated the use of mindfulness techniques [39, 40, 41, 46]; all involved women who had a stillbirth, contained small numbers (*n* = 22–90) and were at moderate or high risk of bias. The interventions included guided mindfulness techniques (combinations of breathing techniques, yoga, body scanning and meditation practices) [39, 40, 41] or yoga alone [46].

Mindfulness techniques were delivered face‐to‐face in group settings [39, 40, 41]. A significant reduction was seen in perinatal grief scores (mean perinatal grief score 109.32 (SD 22.12) at baseline, 92.37 (SD 20.70) at six weeks follow‐up, and 78.16 (SD 24.85) at 1 year follow‐up (*p* = 0.001)) [40] (mean perinatal grief score 110.04 (SD 21.29) at baseline and 93.57 (SD 20.89) at 6‐week follow‐up (*p* = 0.002)) [41] (Table 2 and S1 for all outcome measures).

The yoga intervention was self‐directed online; the control group were given stretching exercises [46]. No significant change was seen in the primary outcome (PTSD symptoms) [46] (Table 2 and S1 for all outcome measures).

The studies show limited evidence that mindfulness techniques had an impact on mental health outcomes and there was a high dropout rate across all interventions [39, 40, 41, 46].

#### 3.5.2 Support Groups

One study (*n* = 46) explored self‐reported use of support groups amongst women who had a stillbirth [50]. Women were recruited online; no details of support group type or setting were available. Women who self‐reported attending support groups had significantly lower traumatic stress response scores than women who did not report attending support groups (mean IES‐R score 3.40 (SD 2.20) vs. 7.56 (SD 1.50; *p* < 0.0001)). Support groups may be of benefit, but a high risk of bias and small sample size affect the validity of results.

#### 3.5.3 Website‐Based Interventions

Three studies evaluated website‐based self‐directed interventions to improve mental health or wellbeing [43, 47, 53]. One study included women who had a stillbirth, and the intervention was adding a closed social media group to a yoga intervention [47]. The intervention did not meet the threshold for effectiveness in helping participants cope with grief.

One study involved parents who had a stillbirth, relatives and healthcare professionals [53] engaging with a web‐based audio archive of recordings of bereaved parents narrating their stories. The website aimed to raise the profile of the psychological and social impact of stillbirth and reduce stigma; 83% of bereaved parents and relatives felt that listening to the archive was very helpful or helpful.

In the final study, women with recurrent miscarriage had access to a website resource plus routine care [43]. The study reported a significant decrease in depression score (mean within group change in Edinburgh Depression score −1.86 (95% CI −3.45, −0.28) intervention group vs. −0.89 (95% CI −2.04, 0.25) control group (*p* = 0.023)) and perceived stress score (mean within group change in Perceived Stress Score −1.86 (95% CI −3.64, −0.08) intervention group vs. control group −1.61 (95% CI −3.25, 0.04; *p* = 0.041)). The limited evidence indicates website‐based interventions may have potential benefit as an adjunct to standard care.

### 3.6 How Effective Are the Interventions?

The heterogeneity of the studies identified and outcome measures, along with a high risk of bias for most studies, meant that it was not possible to determine the most effective interventions to improve a woman's mental health and wellbeing. The review has provided limited evidence that the reported interventions have the potential to improve mental health and wellbeing, but no firm conclusions can be drawn. As identified studies only aimed to improve mental health and wellbeing, it was not possible to determine the most effective interventions to improve the physical health of women or subsequent pregnancy outcomes.

### 3.7 What Were the Experiences of Participants and Were Interventions Acceptable?

Feedback was generally positive in those studies that reported on participants' experiences of an intervention. One study reported treatment satisfaction scores for participants who had CBT or anti‐deressants [45]; participants in the CBT group had a significantly higher satisfaction score compared to the anti‐depressant group (*p* < 0.001).

One study reported on participants' experiences of counselling [49]. Counselling was not always helpful, but when it was helpful it had the following traits: ‘compassionate and understanding’, ‘non‐judgemental’, ‘accepting of parents' emotional state’, ‘deep listening/place for narration and processing emotion’ and ‘gave perspective’. Barriers to access included financial constraints and the need to return to work.

Mindfulness techniques had high levels of acceptability when used as an intervention [39, 40, 41, 46]. ‘*Time constraints’* were reported as a barrier to delivery in both face‐to‐face interventions in rural India and online interventions in the USA [39, 40, 46].

Participants who used support groups reported how ‘helpful interacting with other parents was in honoring, recognizing and destigmatizing their current emotional state’ [50]. Support groups helped with the burden of guilt and recognizing and dealing with their cognitive and emotional state.

All the website‐based interventions reported good acceptability [43, 47, 53]. Participants who used a social media group reported it helped them cope with grief by feeling part of a group and reduced feelings of loneliness [47]. For the online archive 11 respondents reported feeling less alone after listening [53]. Amongst participants who used the website as an adjunct to routine care, a third of participants reported it provided useful information, a third reported it helped to promote stress relief and relaxation and 29% reported sharing experiences could be helpful [43].

### 3.8 What Were the Experiences of Healthcare Professionals?

None of the studies provided feedback from healthcare professionals on their experiences of delivering an intervention. This is an area requiring further research and exploration.

## 4 Discussion

This review aimed to determine the most effective interventions to improve general health and subsequent pregnancy outcomes for non‐pregnant women who had a stillbirth, second trimester miscarriage, or recurrent miscarriage. A significant gap was found in the evidence. No studies identified interventions that could potentially improve the physical or longer‐term health of women or address how best to provide preconceptual care for subsequent pregnancies. This finding is important given recent systematic review evidence demonstrating the increased lifelong cardiovascular and autoimmune conditions in women who have a history of pregnancy loss [55]. Similarly, there was no evidence regarding how to provide care that would improve outcomes in subsequent pregnancies.

When studies had been undertaken, there was still insufficient evidence to determine the most effective interventions. Several interventions addressing mental health and wellbeing, including CBT, counselling, mindfulness techniques, the use of support groups and website‐based interventions, showed potential, but more high‐quality evidence is needed to understand how and when to best deliver care to improve mental health and which of these interventions are most effective. This review also found limited evidence to understand the experiences of women participating in the interventions, the acceptability of the interventions to women and the feasibility of delivering them.

All interventions identified were for women who had a stillbirth or recurrent miscarriage, with none specifically looking at care for women who had a second trimester miscarriage. No studies were identified that specifically looked at care for underserved groups of women in high‐income countries. Barriers to access and disparities in care persist for women from underserved groups. These barriers include language difficulties, unclear or absent access pathways, digital poverty, care located a significant distance from home, discrimination, prejudice and a lack of culturally safe and trauma‐informed care [56, 57, 58, 59]. Understanding how to dismantle these barriers, recognising the intersectionality of these disparities and adapting care to meet the needs of women is essential, as these are the groups who are most likely to experience a pregnancy loss [1, 56]. No studies were identified that included non‐binary or transgender birthing people, a group that may need specialist support and face additional challenges to those experienced by cisgender women [60, 61].

No evidence was identified to suggest an ideal location or type of healthcare provider to deliver care. Typically, a pregnancy loss and immediate care following this often occurs in hospital; women are then discharged back into the community. The provision of community bereavement midwives demonstrates wide geographical variation; obstetricians are often not provided with formal training in care following a pregnancy loss, and primary care services are not a routine part of care [62]. Community services may be ideally placed to deliver care following a pregnancy loss, especially for women who face multiple access barriers or when returning to the hospital where the loss occurred may cause further distress [16]. However, the role of GPs in delivering care after pregnancy loss is largely unexplored [62]. GPs, and the multi‐disciplinary teams they work with in primary care, are a skilled workforce trained in providing holistic care to people with multimorbidity, and their role in care needs further exploration. Further research needs to explore wider methodologies than randomised control trials alone and should be tailored to the outcome and intervention being studied [63]. It is also vital that any future research is co‐produced with women and their families to include their perspectives and experiences and ensure that potential interventions are acceptable to women and meet their needs [62, 64].

### 4.1 Strengths and Limitations

This is the first systematic review specifically focused on interventions delivered after discharge to the community following pregnancy loss. Having systematically reviewed the available evidence, we found that there was insufficient evidence to answer the review question. The evidence was also poorly defined in terms of population and intervention content and delivery. Most studies were at high risk of bias, and the quality of evidence in the included studies was low. There was significant variation in the outcome measures used for reporting, which made it impossible to compare or pool results. This is something that may improve in the future with the advent of the development of a core outcome set for stillbirth research [19] and for mental health following early pregnancy loss [20]. A final limitation of this work is that the searches were limited to English language, which may inhibit the incorporation of results from other settings.

### 4.2 Interpretation

The review has demonstrated that there is a gap in the availability and quality of evidence to determine how to best care for women following stillbirth, second‐trimester miscarriage and recurrent miscarriage. Our findings are consistent with other reviews that have examined similar questions [23, 24]. A Cochrane review in 2018 examined what the most effective care was prior to or during a subsequent pregnancy for improving outcomes in women who have a stillbirth and found there was insufficient evidence to answer the review question [24]. The findings of our review are also consistent with the National Bereavement Care Pathway, which makes no specific recommendations on what care should be provided for women after discharge into the community [13]. Pregnancy loss has a significant impact on women and their families [1, 2, 65], which demonstrably reduces quality of life [66] and has an impact on lifelong health.

## 5 Conclusions

The results of the review demonstrate a large gap in the available evidence to determine how we should best care for women after discharge to the community following a stillbirth, second trimester miscarriage, or recurrent miscarriage. There is an urgent need for research that addresses this evidence gap and determines how best to deliver care that meets the needs of women. Research needs to focus on interventions that provide support, improve physical and mental health and reduce the loss of quality‐adjusted life years irrespective of future pregnancy intention.

## Author Contributions

B.M. conceived the review idea, completed the searches, screened studies and full text articles, extracted data, undertook the narrative synthesis and wrote the paper. S.M. screened studies and full text articles, extracted data and reviewed and revised the paper. H.L. and C.M. screened studies and reviewed and revised the paper. J.D., A.G., A.E.P.H., J.P. and S.S. provided methodological support and reviewed and revised the final draft and subsequent reviews. S.H. screened studies and full text articles, provided methodological support and reviewed and revised the paper.

## Conflicts of Interest

The authors declare no conflicts of interest.

## Supporting information


**Appendix S1:** bjo70043‐sup‐0001‐AppendixS1.docx.

**Appendix S2:** bjo70043‐sup‐0002‐AppendixS2.docx.

**Appendix S3:** bjo70043‐sup‐0003‐AppendixS3.docx.

**Table S1:** Summary table of characteristics of included studies.

## Data Availability

The authors have nothing to report.
