# Supplementary material for: Interventions to Improve Outcomes After Pregnancy Loss: A Systematic Review
Source: BJOG. 2025 Oct 17;133(3):365–74. doi: 10.1111/1471-0528.70043 (PMC12770074; doi:10.1111/1471-0528.70043)
Supplement: Supplementary file 1 — Appendix S1: bjo70043‐sup‐0001‐AppendixS1.docx. [file BJO-133-365-s001.docx]

**Appendix 1 - Search strategy**

| \| **OR**  **🡫** \|  \| **OR**  **🡫** \|  \| **OR**  **🡫** \| \| --- \| --- \| --- \| --- \| --- \| \| ‘fetal demise’  ‘fetal loss’  ‘fetal death’  ‘intrauterine death’  ‘intrauterine fetal death’  ‘stillbirth’  ‘stillbirth*’  ‘second trimester miscarriage’  ‘second trimester pregnancy loss’  ‘second trimester spontaneous abortion’  ‘late miscarriage’  ‘late pregnancy loss’  ‘recurrent miscarriage’  ‘recurrent pregnancy loss’  ‘recurrent spontaneous abortion’ \| **AND** \| MeSH Terms:  Perinatal Care/  Postnatal Care/  Preconception Care/  Prenatal Care  ‘care’  ‘interven*’ \| **AND** \| ‘improv*’‘reduc*’‘prevent*’ \| |
| --- | --- | --- | --- | --- | --- | --- | --- | --- | --- | --- |
