# Supplementary figures and images for: Interventions to Improve Outcomes After Pregnancy Loss: A Systematic Review
Source: BJOG. 2025 Oct 17;133(3):365–74. doi: 10.1111/1471-0528.70043 (PMC12770074; doi:10.1111/1471-0528.70043)

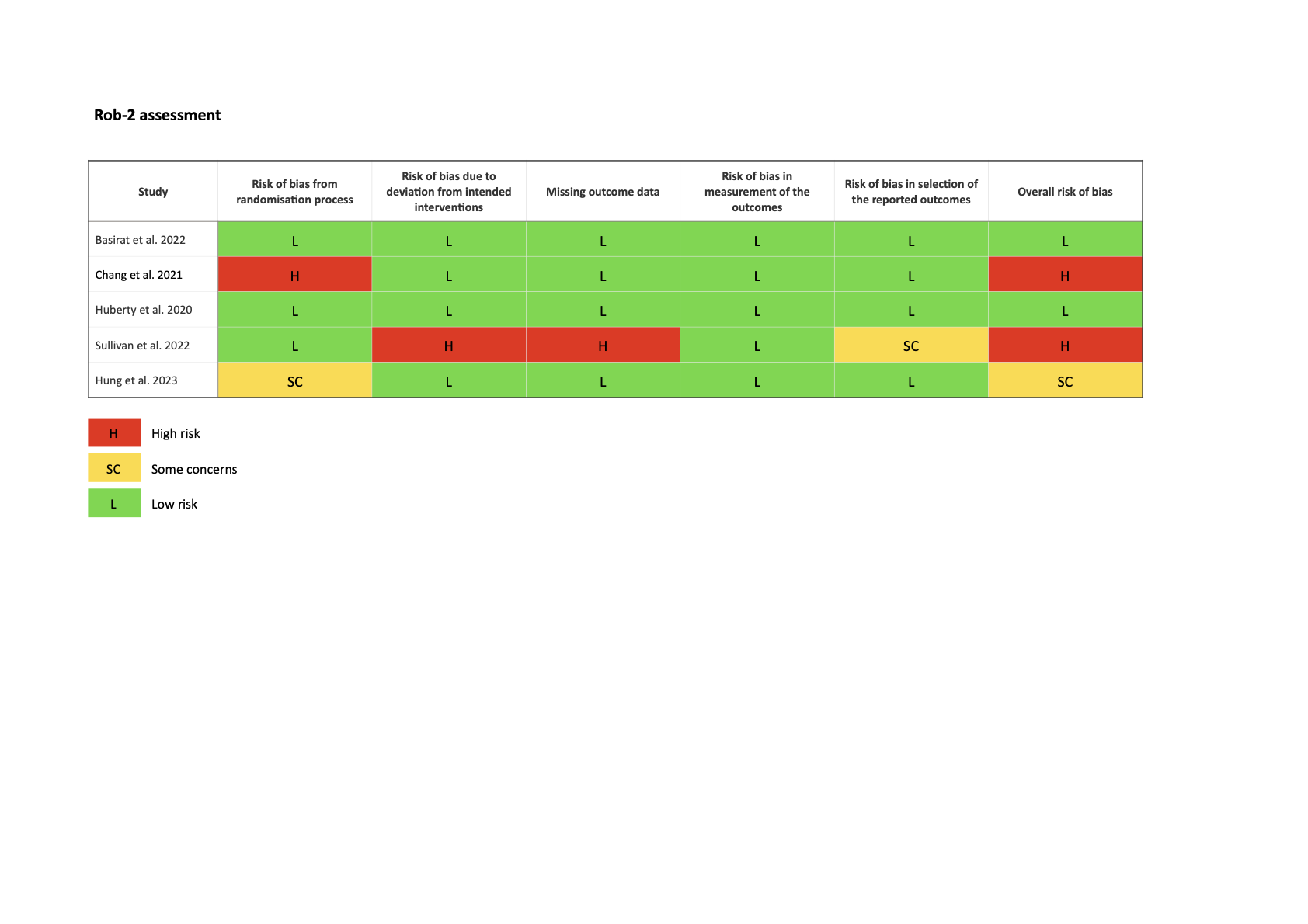

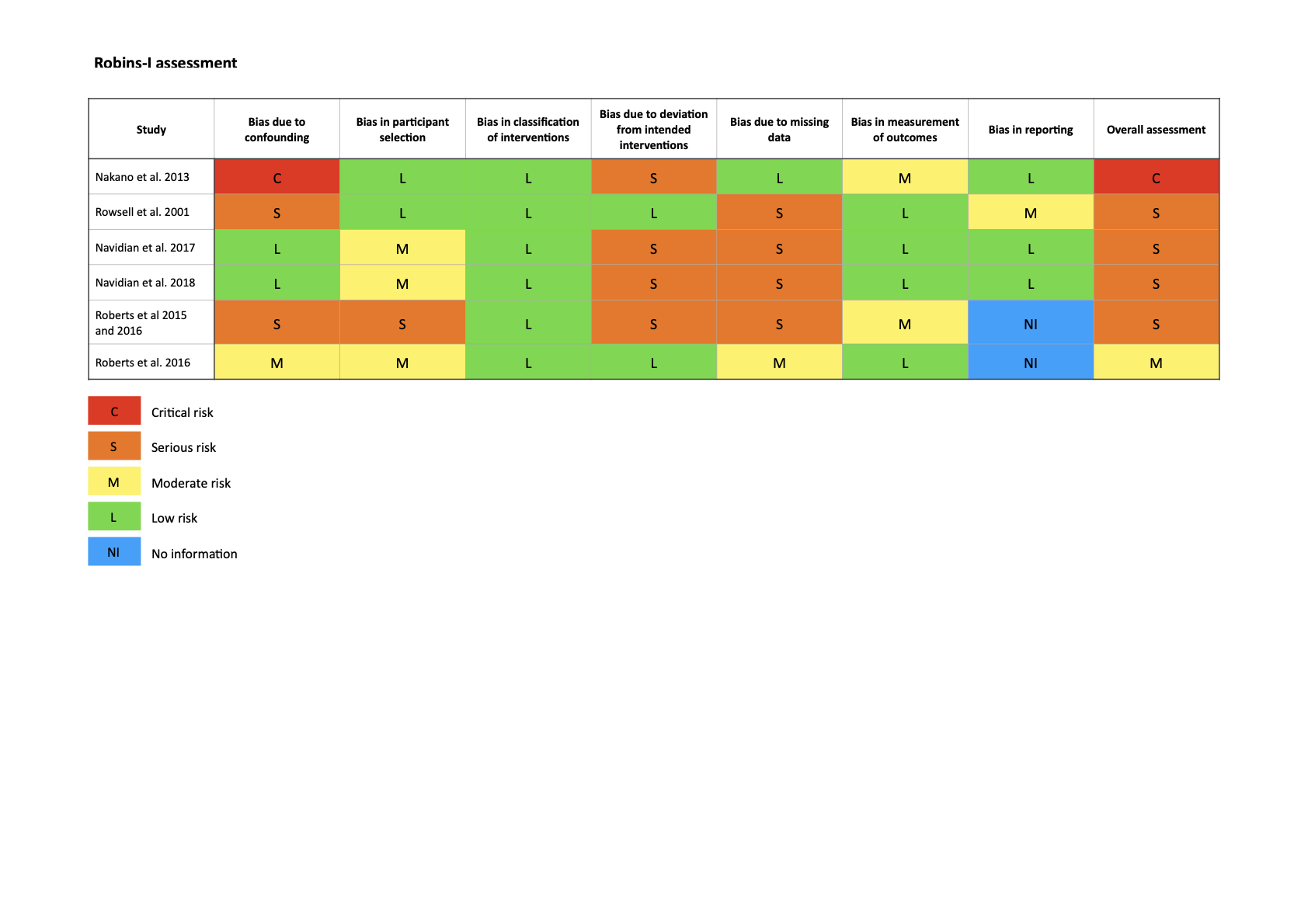


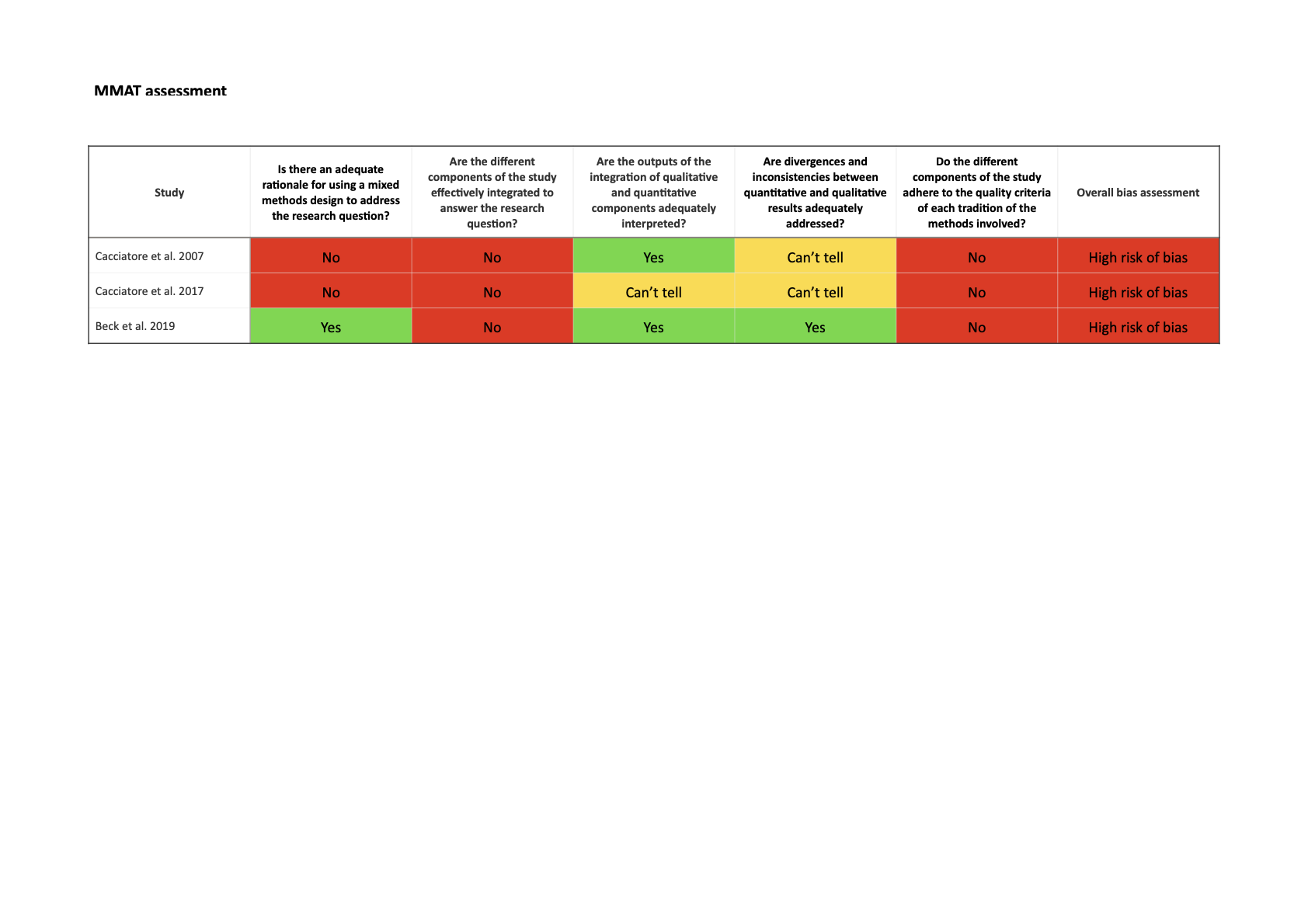

Supplement: Supplementary file 2 — Appendix S2: bjo70043‐sup‐0002‐AppendixS2.docx. [file BJO-133-365-s002.docx]
