# Supplementary material for: Interventions to Improve Outcomes After Pregnancy Loss: A Systematic Review
Source: BJOG. 2025 Oct 17;133(3):365–74. doi: 10.1111/1471-0528.70043 (PMC12770074; doi:10.1111/1471-0528.70043)
Supplement: Supplementary file 3 — Appendix S3: bjo70043‐sup‐0003‐AppendixS3.docx. [file BJO-133-365-s004.docx]

**Record of amendments to protocol**

Samuel Madejowski, Amy Grove, Alexander Heazell and Sophie Staniszewska were added to the protocol as authors.

Inclusion of the Cochrane Review database in the search strategy was removed because when this was searched as part of the initial test only systematic reviews were identified.

The inclusion and exclusion criteria were further clarified to identify the interventions that the review was aiming to capture.

Review progress sections have been updated as the review has progressed.
