# Supplementary material for: Interventions to Improve Outcomes After Pregnancy Loss: A Systematic Review
Source: BJOG. 2025 Oct 17;133(3):365–74. doi: 10.1111/1471-0528.70043 (PMC12770074; doi:10.1111/1471-0528.70043)
Supplement: Supplementary file 4 — Table S1: Summary table of characteristics of included studies. [file BJO-133-365-s003.docx]

| Study | Location | Design | Population | Intervention | Outcome measures | Findings |
| --- | --- | --- | --- | --- | --- | --- |
| Nakano et al. 2013 ^42^ | Japan | Pilot study | Women who have had recurrent miscarriages (n = 14) | Individual Cognitive Behavioural Therapy (CBT)  No comparator | - Depression - Beck Depression Score - Anxiety - State-Trait Anxiety Inventory-state Score | - Significant reduction in depression score at the end of the intervention (p 0.001)  - Significant reduction in anxiety score at the end of the intervention (p 0.016) |
| Basirat et al. 2022 ^45^ | Iran | Triple-arm randomised control trial | Women who have had recurrent miscarriages (n = 60) | Group CBT (n=20) vs Sertraline (n=20) vs Routine care (n=20) | - Depression - Beck Depression Score - Anxiety - State-Trait Anxiety Inventory-state Score - Treatment satisfaction - Treatment compliance | - Significant reduction in depression score with CBT at end of intervention (Hedges’ g 0.84 (95% CI 0.30-1.37) and follow up (Hedges’ g=0.54 (95% CI 0.14-1.11)  - Significant reduction in depression score with sertraline at end of intervention (Hedges’ g=1.03 (95% CI 0.53-1.53) but not at follow up (Hedges’ g = 0.04 (95% CI -0.29-0.38)  - No significant reduction in anxiety score with CBT at the end of the intervention or at follow up  - Significant reduction in anxiety score with sertraline at end of intervention (Hedges’ g=1.21 (95% CI 0.58-1.84) and at follow-up (Hedges’ g=0.83 (95% CI 0.31-1.35)  - Treatment satisfaction was significantly higher in the CBT group compared to sertraline or usual care (p<0.001)  - Dropout rates were 5% in the CBT group, 35% in the sertraline group and 10% in the usual care group. |
| Rowsell et al. 2001 ^48^ | UK | Within-subject repeated measures study | Women who have had recurrent miscarriages (n = 37) | Individual Counselling  No comparator | - Anxiety and depression – Hospital Anxiety and Depression Scale (HADs) - Intrusive thoughts and avoidance - Impact of Event Scale (IES) - Response strategies - Coping Schedule | - Anxiety score significantly reduced (p <0.05) over time from beginning to end of intervention (mean 6.7 weeks)  - Depression score significantly reduced (p < 0.01) over time from beginning to end of intervention (mean 6.7 weeks)  - Avoidance score significantly reduced (p <0.001) over time from beginning to end of intervention (mean 6.7 weeks)  - Intrusive thoughts measured by IES significantly reduced (p <0.001) over time from beginning to end of intervention (mean 6.7 weeks)  - 76 participants started the intervention, but only 37 completed the intervention and were included in analysis |
| Cacciatore et al. 2017 ^49^ | USA | Mixed methods cross-sectional survey | Women who have had a stillbirth  (n = 103) | Counselling  No comparator | - Views of women of the counselling they received after a stillbirth. | - 59% of respondents reported counselling was *very helpful* or *helpful,* 17% *somewhat helpful*, 23% *unhelpful* or *very unhelpful*.  - Helpful counselling had five main themes, compassionate and understanding, non-judgemental, accepting of parent's emotional state, deep listening/place for narration and processing emotion and perspective  - Unhelpful counselling had five themes: lacking compassion and understanding, judgemental, hurtful remarks/platitudes, not accepting of emotional state, attempting to find forced meaning in parent's loss.  - Barriers mentioned were return to work and financial costs |
| Navidian et al. 2017 ^51^ | Iran | Semi-experimental | Women who have had a stillbirth >22 weeks (n = 100) | Group Counselling (n=50) vs routine care (n=50) | - Post-traumatic stress symptoms - Prenatal Posttraumatic Stress Questionnaire | - Mean post-traumatic stress symptom severity score significantly reduced in counselling group vs control group (p 0.001) |
| Navidian et al. 2018 ^52^ | Iran | Semi-experimental | Women who have had a stillbirth >22 weeks (n = 100) | Group Counselling (n=50) vs routine care (n=50) | - Grief severity – Short version of the Perinatal Grief Scale | - Mean grief severity score was significantly reduced in counselling group vs control group (p 0.0001) |
| Chang et al. 2021 ^44^ | Taiwan | Single-blind RCT | Women who have had recurrent miscarriages (n = 62) | Individual Counselling (n=31) vs routine care (n=31) | - Sleep quality - Pittsburgh Sleep Quality Index - Depression - Edinburgh Prenatal Depression Score - Perceived stress - Perceived Stress Score - Level of social support - Interpersonal Support Evaluation List | - Significant reductions in perceived stress score (p 0.028) and depression score (p 0.037)  - 62% participants reported the intervention sessions promoted self-awareness and 38% reported feeling empathetic companionship and stress-relief relaxation  - Dropout rates were 3% in intervention group and 9.7% in routine care group |
| Roberts et al. 2015 ^39^ | India | Pilot study | Women who have had a stillbirth >28 weeks (n = 22) | Group Mindfulness  No comparator | - Depression - Hopkins Symptoms Check List-10 - Life satisfaction - Satisfaction with Life Scale - Grief severity - Perinatal Grief Scale - Social support - Social Provisions Scale - Mindfulness - Five Facet Questionnaire Short Form | - Significant increase in mindfulness across 4 domains of the Five Facet Questionnaire; describe (p 0.02), non-judge (p 0.031), observe (p 0.015) and act aware (p 0.01)  - Significant increase in perceived social support score (p 0.031)  - Intervention not feasible in this population due to daily lives.  - Dropout rate was 77% and attributed mainly to time constraints |
| Roberts et al. 2016 ^40^ | India | Pilot study follow up at 12 months | Women who have had a stillbirth >28 weeks (n = 22) | Group Mindfulness  No comparator | - Depression - Hopkins Symptoms Check List-10 - Life satisfaction - Satisfaction with Life Scale - Grief severity- Perinatal Grief Scale - Social support - Social Provisions Scale - Mindfulness - Five Facet Questionnaire Short Form | - Significant reduction in perinatal grief score (p 0.001) and depression symptoms (p 0.001) at 1 year follow up  - 21 participants were still practising mindfulness at 1 year follow up  - All participants gave positive feedback; majority reported mindfulness gave a feeling of peace |
| Roberts et al. 2016 ^41^ | India | Pilot study | Women who have had a stillbirth  (n = 29) | Group Mindfulness  No comparator | - Depression - Hopkins Symptoms Check List-10 - Life satisfaction - Satisfaction with Life Scale - Grief severity - Perinatal Grief Scale - Religious coping – Short form Brief RCOPE - Social support - Social Provisions Scale - Mindfulness - Five Facet Questionnaire Short Form | - Significant reductions in perinatal grief score (p 0.002)  - Dropout rate was 20%  - All participants said they would continue to use mindfulness techniques after the end of the study  - All participants gave positive feedback indicating that they thought it was useful and made them feel good |
| Huberty et al. 2020 ^46^ | USA | Three group randomised feasibility trial | Women who have had a stillbirth >20 weeks (n = 90) | Online Yoga Sessions: Moderate dose yoga (n=30) vs. low dose yoga (n=30) vs. stretching (n=30) (control group) | Primary outcome:   - Post-traumatic stress disorder symptoms - Impact Event Scale   Secondary outcomes:   - Depression - PHQ-9, - Grief severity - Perinatal Grief Scale, - Self-compassion - Self-Compassion Scale - Emotion regulation - Emotion Regulation Questionnaire - Mindfulness - Mindfulness Attention Awareness Scale - Sleep quality - Pittsburgh Sleep Quality Index - Anxiety - State-Trait Anxiety Inventory | - No significant change in the primary outcome measure  - There was a significant reduction in depression scores in the moderate and low dose yoga groups compared with the stretching group (p 0.036)  - There was a significant reduction in grief severity scores in the moderate and low dose yoga groups compared with the stretching group (p 0.009)  - Dropout rates were 47% in moderate dose yoga, 40% in low dose yoga and 53% in stretching attributed mainly to time constraints  - 64% of participants who completed the intervention said they enjoyed it |
| Cacciatore et al. 2007 ^50^ | USA | Cross-sectional survey | Women who have had a stillbirth >20 weeks (n = 46) | Support Group Attendance (n=29) vs No Support Group Attendance (n=17) | - Traumatic stress response - Impact of Events Scale- Revised (IES-R) - Participants views of attending support groups | - Women who self-reported attendance at support groups had a significant reduction in IES-R score p= <0.0001  - Women reported groups helped with burden of grief, helped with recognising and dealing with cognitive and emotional state  - Support groups may provide safety netting and connections to help with feelings of trauma and isolation |
| Beck et al. 2019 ^53^ | UK | Mixed-methods survey | - Parents who have had a stillbirth and their relatives (n = 33)  - Clinicians caring for parents who have had a stillbirth (n=46) | Website – online archive of stillbirth stories  No comparator | Frequency reported emotions: anger, confusion, fear, anxiety, guilt, isolation, shame, proud, pessimism, acceptance, content informed, confident, supported, optimistic, coping | - 54% families found the stories very helpful and 29% helpful  - 54% of families would use again  - 64% families would signpost the website to other families  - Among parents and relatives ‘feeling supported’ increased from 12% to 27%, ‘feeling alone’ decreased from 27% to 15%, ‘anxiety’ decreased from 27% to 12%  - 72% clinicians felt they had good understanding of the emotional impact of stillbirth  - 12 clinicians gave feedback after watching the archive with all reporting they had learnt something new  - After listening to archive 7 clinicians felt either very confident or confident in being equipped to meet families’ emotional needs |
| Sullivan et al. 2022 ^47^ | USA | Randomised parallel feasibility trial | Women who have had a stillbirth  (n = 60) | Website – Facebook support group added to a yoga intervention (n=30) vs no Facebook group added to a yoga intervention (n=30) | Feasibility:   - Demand – measured in minutes per week of yoga, minutes per week on Facebook and Facebook Analytics - Feasibility - measured by a researcher designed questionnaire covering 13 questions assessing satisfaction with the intervention   Psychosocial outcomes:   - Traumatic stress response - Impact of Events Scale- Revised (IES-R) - Anxiety - State-Trait Anxiety Inventory - Depression - PHQ-9 - Support seeking - Berlin Social Support Scale | - >70% of participants reported >75% satisfaction for 8 of the 13 questions assessing feasibility - No significant differences in minutes of yoga per week between participants with access to the Facebook group and participants without access to the Facebook group  - Dropout rates were 57% in the Facebook group and 37% in the group without Facebook  - No significant difference in the psychosocial outcome measures  - Participants did not meet benchmark for the Facebook group helping ability to cope with grief |
| Hung et al. 2023 ^43^ | Taiwan | Single-blind randomised control trial | Women who have had recurrent miscarriages (n = 62) | Website – access to a mental health website intervention with routine care (n=31) vs routine care (n=31) | - Sleep quality - Pittsburgh Sleep Quality Index - Depression - Edinburgh Prenatal Depression Score - Perceived stress - Perceived Stress Score - Level of social support - Interpersonal Support Evaluation List | - Significant reduction in depression score (p 0.023) and perceived stress score (p 0.041)  - Dropout rates were 6% in the intervention group and 10% in the control group  - 8 participants reported website was useful - 7 participants reported sharing experiences as helpful |

Table 2. Summary table of characteristics of included studies
